# Application of new biotechnologies for improvements in swine nutrition and pork production

**DOI:** 10.1186/s40104-019-0337-6

**Published:** 2019-04-19

**Authors:** Guoyao Wu, Fuller W. Bazer

**Affiliations:** Department of Animal Science and Center for Animal Biotechnology and Genomics, Texas A&M University, College Station, TX 77843-2471 USA

**Keywords:** Biotechnology, Disease, Growth, Health, Meat production, Swine

## Abstract

Meeting the increasing demands for high-quality pork protein requires not only improved diets but also biotechnology-based breeding to generate swine with desired production traits. Biotechnology can be classified as the cloning of animals with identical genetic composition or genetic engineering (via recombinant DNA technology and gene editing) to produce genetically modified animals or microorganisms. Cloning helps to conserve species and breeds, particularly those with excellent biological and economical traits. Recombinant DNA technology combines genetic materials from multiple sources into single cells to generate proteins. Gene (genome) editing involves the deletion, insertion or silencing of genes to produce: (a) genetically modified pigs with important production traits; or (b) microorganisms without an ability to resist antimicrobial substances. Current gene-editing tools include the use of zinc finger nuclease (ZFN), transcription activator-like effector nuclease (TALEN), or clustered regularly interspaced short palindromic repeats-associated nuclease-9 (CRISPR/Cas9) as editors. ZFN, TALEN, or CRISPR/Cas9 components are delivered into target cells through transfection (lipid-based agents, electroporation, nucleofection, or microinjection) or bacteriophages, depending on cell type and plasmid. Compared to the ZFN and TALEN, CRISPR/Cas9 offers greater ease of design and greater flexibility in genetic engineering, but has a higher frequency of off-target effects. To date, genetically modified pigs have been generated to express bovine growth hormone, bacterial phytase, fungal carbohydrases, plant and *C. elagan* fatty acid desaturases, and uncoupling protein-1; and to lack myostatin, α-1,3-galactosyltransferase, or CD163 (a cellular receptor for the "blue ear disease" virus). Biotechnology holds promise in improving the efficiency of swine production and developing alternatives to antibiotics in the future.

## Introduction

**Fig. 1:**
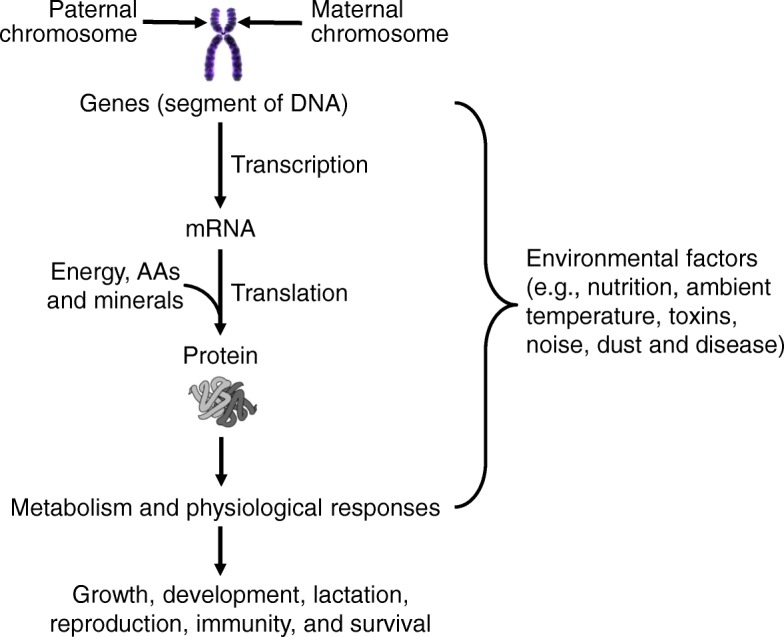
Role of genes in the growth, development, lactation, reproduction, and health of swine. The domesticated pig has 19 pairs of chromosomes (a total of 38 chromosomes), with one set of chromosomes from each parent. A chromosome contains segments of the DNA molecule that are called genes. A trait is controlled by two variant forms of a gene (called an allele) located at the same position (genetic locus) in the pair of chromosomes, with one allele inherited from each parent. Expression of genes through the transcription and translation processes to produce proteins is affected by environmental factors (including nutrition, ambient temperature and disease) in a cell-specific manner

Pork provides high-quality animal protein for human consumption, and is a popular food in China and many other countries, including the United States, Canada, Japan, and Europe. As for any livestock species, the production performance (e.g., growth rate, feed efficiency, litter size, and meat quality) of swine depends on genes (functional units along the DNA molecule, genetic materials), environmental factors (e.g., nutrition, ambient temperature, toxins, and disease), and their interactions (Fig. 1). Genes (genotypes) are the basis for the growth, lactation, reproduction, and other production traits of swine. However, genes can be optimally expressed to yield desirable phenotypes only under favorable environmental conditions, such as optimal provision of dietary nutrients (energy, amino acids, fatty acids, carbohydrate, vitamins, and minerals), desirable ambient temperatures, high-quality air, and clean drinking water.

A major goal of pork production is to fully realize the genetic potential of swine for reproduction, lactation, growth (including accretion of protein in skeletal muscle), and resistance to disease, while preventing excessive accumulation of white adipose tissue and reducing the excretion of wastes (nitrogen and minerals) into the environment [1]. Over the past 60 years, efficiency of pork production has been improved tremendously due to advances in animal breeding, nutrition and management. For example, Boyd and Cady [2] reported that in the United States, the feed:gain ratio (kg feed consumed/kg dressed carcass) of growing pigs was reduced by 33% from 6.6 in 1959 to 4.4 in 2009 and that the carbon footprint (kg CO_2_e/lb. of dressed carcass) was reduced by 35% over this 50-year period. However, the swine industry still faces many challenges. First, high rates of embryonic losses, intrauterine growth restriction, and preweaning mortality occur in swine [3]. Second, dietary energy is readily partitioned toward the spontaneous and rapid accumulation of subcutaneous white adipose tissue in growing-finishing pigs [4]. Third, swine have a suboptimal ability to digest plant-source protein, minerals and fibers. Fourth, swine (particularly during gestating, lactating, growing-finishing, and breeding periods) exhibit a high susceptibility to heat stress and infectious disease. Fifth, alternatives to feed antibiotics are urgently needed in swine production [5]. Thus, enhancing the efficiency of feed utilization and reducing production costs are continually required to increase the profitability of the global pork industry. An important approach to solving these problems is the use of new biotechnologies, including cloning, genetic engineering (producing transgenic animals), gene editing, production of vaccines, and microbial fermentation of feedstuffs.

## Basic concepts of chromosomes, genes and alleles related to animal biotechnology

Knowledge of the cell, which is the basic unit of the animal, plant and microorganism, is necessary for understanding biotechnologies. Animal cells and bacteria contain the plasma membrane, cytoplasm, and nucleus, while most animal cells also contain mitochondria. The conversion of dietary nutrients into biological energy in animals requires both the cytoplasm and the mitochondria (the major powerhouse), whereas this process occurs only in the cytoplasm via glycolysis in bacteria. Energy supply is vital for cell integrity and function. The nucleus is the site of: (a) DNA synthesis and storage; (b) chromosomes (a carrier of long DNA molecules and associated proteins); (c) DNA-directed RNA synthesis; and (d) control of protein synthesis and cell growth. Thus, this organelle is highly significant for the development of new biotechnologies to alter the production of proteins (including enzymes and cellular components) and peptides (including vaccines and antimicrobials).

The domesticated pig has 38 chromosomes arranged in 19 pairs (2 chromosomes per pair, with one chromosome from each parent), including one pair of chromosomes called sex chromosomes (XX for females and XY for males). Each chromosome contains different segments (units of inheritance, genes) of DNA for the cell to synthesize proteins, thereby controlling the functioning of the organism. The double-helix DNA molecules consist of deoxyribose sugars, phosphates, and nitrogenous bases [adenine (A), cytosine (C), guanine (G), and thymine (T)] organized in pairs (A-T and G-C) via ion bonds. The whole set of chromosomes for the pig contains all of its genes (called the genome). A trait (or characteristic) is controlled by two variant forms of a gene (called an allele) located at the same position (genetic locus) in the pair of chromosomes, with one allele inherited from each parent. Dominant and recessive alleles are the determinants for a single trait. This provides the basis for the conservation of genetic materials in pigs and the insertion of a new gene into a pig.

A quantitative trait locus (QTL) is a section of DNA that correlates with variation in the phenotype of a population of organisms (e.g., growth rate, litter size, and milk yield). QTLs are mapped by identifying which molecular markers [such as single nucleotide polymorphisms (SNPs)] correlate with an observed trait. A gene marker is a sequence of DNA that is linked to a gene affecting a trait, and is of economic importance in swine production. For example, Andersson et al. [6] reported the presence of a QTL on chromosome 4 for controlling growth from birth to 70 kg, the length of the small intestine, and fat deposition in pigs (European wild boar × Large White cross). In addition, a QTL on pig chromosome 8 was identified as secreted phosphoprotein-1 for prenatal survival and litter size [7], and this protein is now known to regulate ion and water transport by the pig placenta [8]. To date, with the RNA-Seq (RNA sequencing; also called next generation sequencing) technology, genomics biology has extended beyond the traditional genomic sequencing to defining the entire transcriptome of an organism [9]. In research, the RNA-Seq can be used to determine the presence and quantity of RNA (including mRNA, tRNA, and micro RNA) in a biological sample. This revolutionary method is now used increasingly to analyze gene expression and discover SNPs in animals. Genomes that are resistant or susceptible to certain diseases, or animals that have high versus low production performance (e.g., milk yield, muscle growth rate, feed efficiency) can be compared and identified, with the goal to improve the health and production performance of swine at different stages of their life cycle.

## Biotechnologies in swine nutrition and production

By definition, biotechnologies are technologies used in biological research and applications. In a broad term, biotechnologies can be classified as cloning of animals (via embryonic cell nuclear transfer and somatic cell nuclear transfer) and genetic engineering [recombinant DNA (rDNA) technologies, gene editing, and production of transgenic animals] [10]. Cloning of animals refers to the production of genetically identical individuals through asexual reproduction for the conservation of genetic materials. This can occur naturally in the birth of identical twins. Cloning can also be accomplished via embryo spitting by transferring up to four single blastomeres from a 4-cell embryo into four different recipient mothers. In contrast, biotechnologies that add, remove, or rearrange DNA to modify phenotypes are called genetic engineering or gene transfer. Cloning and genetic engineering are two different techniques, but can be combined to produce individual animals (e.g., genetically modified pigs with α-1,3-galactosyltransferase gene-knockout for organ transplantation).

## Cloning of animals

### Procedures

**Fig. 2:**
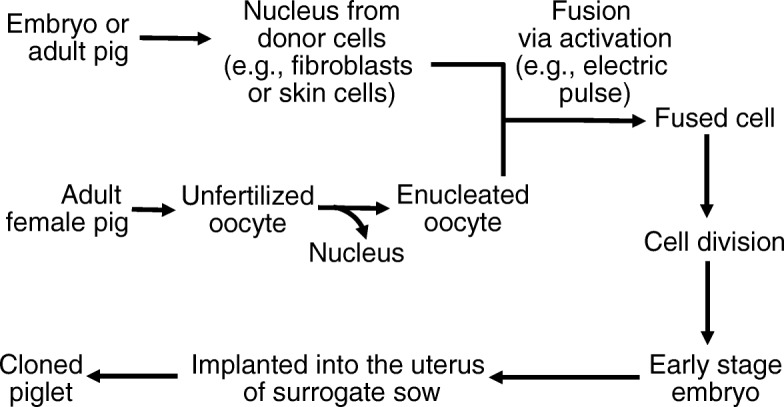
Scheme of pig cloning from embryonic or adult donor cells. An unfertilized, enucleated oocyte from an adult female pig is fused with the nucleus from an embryonic or adult donor cell via electric pulse to form a new cell. This new cell undergoes division in a test tube (culture medium) into an early stage embryo, which is implanted into the uterus of a sow. Ultimately, the sow gives birth to piglets that have the same genetic makeup as the animal that donated the embryonic or somatic cell

The most common use of the term “cloning” refers to the production of an animal from its own cells. When donor cells (e.g., fibroblasts) are from early stage embryos, cloning is known as embryonic cell nuclear transfer (ECNT). For example, Prather et al. [11] reported the first cloning of pigs through the use of embryonic cells as a donor. When donor cells (e.g., skin cells) are from fetuses, young or mature animals, cloning is called somatic cell nuclear transfer (SCNT). Essentially, cloning involves the transfer of a nucleus from a donor cell into a mature enucleated oocyte (an oocyte whose own nucleus has been removed) (Fig. 2). In practice, nuclear transfer can be accomplished by: (a) fusion (micromanipulation of nucleus into the perivitelline space inside the zona pellucida, followed by electrofusion of the two cells); (b) direct microinjection of the nucleus, nucleus plus part of the cytoplasm, or even the entire donor cell into the recipient oocyte; or (c) removal of the zona pellucida from the recipient oocyte by micromanipulation or enzymatic methods, followed by fusion of the donor cell through chemical or electrical methods [10]. In either way, the oocyte develops into an early-stage embryo in the culture plate and then is implanted into the uterus of an adult female. Ultimately, the adult female gives birth to an animal that has the same genetic makeup as the animal that donated the nucleus from an embryonic or somatic cell. This young animal is referred to as a clone when derived from genetically identical parental cells.

Animal scientists have approximately 40 years of experience with cloning. The year of 1979 witnessed the first production of genetically identical mice by splitting mouse embryos in the test tube and then implanting the resultant embryos into uteri of adult female mice. After 276 attempts, animal scientists at a Scottish institute succeeded at employing the SCNT method to produce a healthy lamb called Dolly from cells of the mammary gland of a 6-year-old sheep in 1996 [12]. By directly injecting nuclei from porcine fetal fibroblasts into enucleated oocytes, followed by development induced through electroactivation, Onishi et al. [13] transferred 110 cloned embryos to four surrogate sows, resulting in the birth of one apparently normal female piglet. In addition, Polejaeva et al. [14] were able to generate cloned pigs from adult somatic cells through the nuclear transfer method.

Using the SCNT technique, Betthauser et al. [15] produced four healthy male piglets from two surrogate sows. Of note, genetically identical animals may not be phenotypically identical for either naturally born offspring or the progenies that are produced by cloning [10]. This is mainly because epigenetic factors and environmental factors (e.g., nutrition, ambient temperatures, and air quality) influence gene expression in cells [16].

### Advantages and applications

A clear advantage of cloning is to conserve breeds or species (particularly those that are endangered), thereby maintaining or increasing genetic diversity in the population [10]. Cloning can also allow castrated male animals that possess good traits (e.g., high quality meat, as well as unusually high rates of lean tissue growth and feed efficiency) to pass their genetic traits to offspring. In animal agriculture, the main use of cloning is to produce breeding stock. The United States Food and Drug Administration [17] published an article stating that “meat and milk from clones of cattle, swine (pigs), and goats, and the offspring of clones from any species traditionally consumed as food, are as safe to eat as food from conventionally bred animals”. As noted previously, there is growing interest in cloning pigs to provide special organs for transplantation into human patients with certain diseases [18]. Thus, cloning is useful for not only biomedical and agricultural research, but also the production of pharmaceuticals and natural genetic conservation.

### Disadvantages

The SCNT remains a technically difficult and costly procedure [19]. Major disadvantages of animal cloning are a very low efficiency to produce offspring, as well as poor health and a low survival rate of offspring. This may result from: (a) inappropriate reprogramming of the donor nuclear DNA to metabolically normal phenotypes; and (b) inappropriate interaction between the embryo/fetus and the uterus of the recipient mother. Of note, in swine and cattle, only 1% to 20% of pregnancies continue to term; 25% of the pregnant recipients develop hydrops (with hydrops fetalis being a condition in the fetus characterized by an accumulation of fluid, or edema, in at least two fetal compartments; hydrops allantois or hydrops amnion being an accumulation of excessive fluid within the allantoic or amniotic membranes, respectively); 20–25% of offspring have developmental abnormalities; and high rates of neonatal mortalities (e.g., 30–40% of calves die before 150 days of age) [10, 13, 15]. Although the process of cloning is straightforward, the results are not always predictable due to a variety of complex factors involving cell fusion, embryonic development, and maternal uterine function. Because of its low efficiency, cloning is unlikely to be useful for economic production of a large quantity of meat for human consumption.

## Genetic engineering

### Recombinant DNA technology

**Fig. 3:**
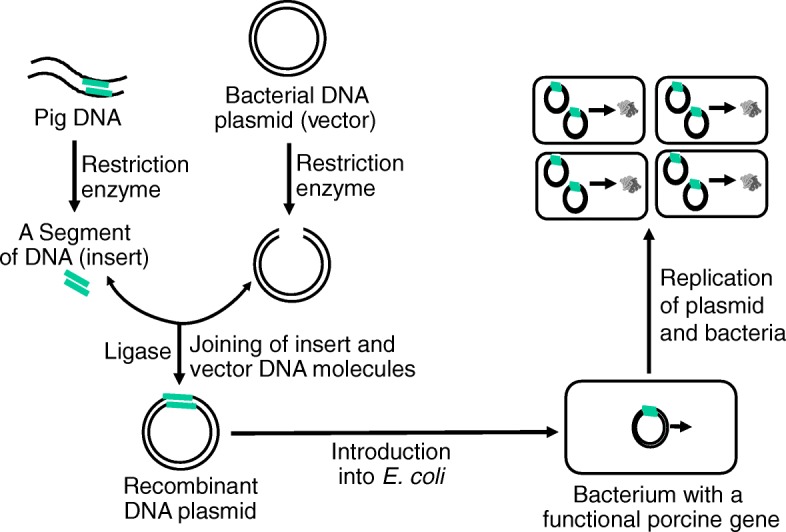
Recombinant DNA technology. With the action of restriction enzymes, a segment of DNA (insert) is isolated from a pig DNA and a bacterial DNA plasmid is cleaved. Catalyzed by a ligase, a DNA insert joins the open plasmid to create a recombinant DNA plasmid, which is then introduced into *E. coli* to produce a protein or polypeptide of interest. The plasmid and bacteria replicate rapidly to generate a large amount of the protein or polypeptide

An rDNA molecule is a DNA molecule formed through laboratory methods from genetic materials derived from two or more sources [20]. The DNA sequences used in the construction of rDNA molecules can originate from any species (including bacteria, plants and animals). The rDNA molecules are sometimes called chimeric DNA, because they can be often made of genetic material from two different species (e.g., pig and bacteria). The rDNA technology differs from genetic recombination in that the former results from artificial methods in a test tube, whereas the latter is a normal biological process leading to the remixing of existing DNA sequences in cells. The basic strategy of rDNA technologies is to insert a DNA fragment of interest (e.g., a porcine DNA) into a vector carrier (a DNA molecule or a plasmid) that is capable of independent replication in a host cell (e.g., *E. coli*). Besides plasmids (circular DNA molecules that originated from bacteria), other most commonly used vectors (non-chromosomal DNAs) are viruses, and yeast cells [21–23]. Inside the host cell (e.g., *E. coli*.), the rDNA carrying a pig DNA insert can replicate rapidly along with the bacteria to generate millions of copies of the plasmid DNA, which directs the synthesis of a protein or polypeptide of interest (Fig. 3).

### Advantages and applications

The rDNA technology offers many advantages. For example, it can modify a single gene locus possibly without perturbing the remainder of the genome and is of great value to basic research, medicine, and agriculture. This biotechnology is the foundation for producing transgenic animals (including pigs; see the section below) [24]. In addition, scientists can use rDNA technologies to produce proteins (including interferon tau, hormones and feed enzymes), peptides, vaccines, amino acids, fatty acids, and vitamins by bacteria, such *E. coli* [25–35], as summarized in Table 1. The costs are low and benefits are enormous. For example, the availability of feed-grade amino acids can substantially reduce the content of protein in diet, thereby decreasing the excretion of nitrogen into the environment. A reduction in dietary protein content by a 1% unit (e.g., from 16% to 15% crude protein) can decrease the excretion of total nitrogen (in urine plus feces) from growing pigs by 8.5% [36]. In addition, rDNA technologies can also modify bacterial genomes to: (a) generate enzymes for feed fermentation [34]; (b) eliminate bacterial resistance to antibiotics by producing enzymes to remove the mediating molecules [35]; and (c) develop vaccines through separation of protein antigens using specific monoclonal antibodies, synthesis of protein antigens by cloned genes, and synthesis of peptides to be used as vaccines [37].

**Table 1:** The use of recombinant DNA technology in producing proteins, vaccines, amino acids and vitamins by bacteria

Product	Function	Reference
Porcine growth hormone (somatotropin)	Enhances lean tissue growth	Chung et al. [25]
Human insulin	Regulates metabolism; treats diabetes	Keen et al. [26]
Vaccines	Prevents bacterial and viral diseases	CAST [27]
Antibodies	Controls viruses (e.g., African swine virus)	CAST [27]
Phytases	Hydrolyzes phytate in plants; increases the digestion of minerals and proteins in diets	Pandey et al. [28]
Carbohydrases	Hydrolyzes carbohydrates in diets	Rosano and Ceccarelli [29]
Feed enzymes with high optimal hydrolyzes dietary carbohydrates Adrio and Demain temperatures	Hydrolyzes dietary carbohydrates and proteins in diets	Adrio and Demain [21]
Antimicrobials	Kill pathogenic bacteria; enhances animal growth and feed efficiency	Diez et al. [30]
Amino acids (e.g., Arg, Glu, Gln, Lys, Thr, and Trp)	Enhances animal growth and feed efficiency	Ma and Chen [31]
Vitamins (both water- and lipid-soluble)	Enhances animal growth and feed efficiency	Vandamme et al. [32]
Enzymes for feed fermentation	Digests complex carbohydrates and proteins; produces small peptides and amino acids	Opazo et al. [33] Demirci et al. [34]
Enzymes for degrading AMR mediators	Enhances animal growth and feed efficiency	da Costa et al. [35]

*AMP* Antimicrobial resistance

### Disadvantages

Because of inadequate research, some people are concerned about the biosafety of proteins (e.g., recombinant bovine growth hormone) or potential by-products generated by the rDNA technology. Insertion of a gene into, or deletion of a gene from, the animal genome may affect the function or stability of genes existing in the organisms [37]. Finally, in vitro culture conditions may not be optimal for high rates of transcription and translation of recombinant genes in the new cells. More work is required to address these important issues.

## Genetically modified animals

**Fig. 4:**
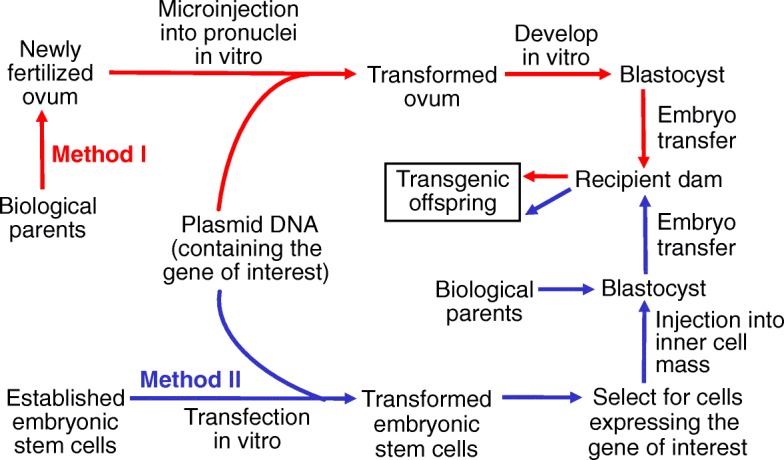
Production of transgenic animals via the injection of the recombinant DNA into the pronucleus of a fertilized ovum (Method I) or the injection of transformed embryonic stem cells that contain the recombinant DNA into a blastocyst (Method II). In the first and most common method used for livestock species, an ovum is surgically collected shortly after its fertilization, and a recombinant DNA (e.g., the plasmid DNA of interest) is then microinjected through a very fine needle into the pronucleus of the fertilized ovum. The transformed ovum is developed into a blastocyst in vitro and the embryo is then transferred into a surrogate mother for development to term. In the second method, established embryonic stem cells (prepared from a preimplantation embryo) expressing the gene of interest in their genome are transfected with a recombinant DNA. Stably transformed ES cells are selected and then injected into the inner cell mass of a recipient blastocyst. After a short period of culture, the embryo that contains the recombinant gene in its genome is transferred into a surrogate mother for development to term. In both methods, surrogate mothers produce transgenic offspring, but the origin of the blastocyst differs

Germ-line and non-germ line transgenic animals can be produced by using new biotechnologies. Ectopic DNA transfer (non-germ line transgenic) refers to the direct administration of DNA constructs or transgenic stem cells into non-reproductive tissues of fetuses or living animals to yield transgenic animals, but their transgenic traits are not passed onto future generations via the gametes [10]. Germ-line transgenesis will be the focus of this article (Fig. 4). In essence, the production of transgenic animals is based on biochemical reactions, cell biology, cell culture, embryo transfer, and fetal growth and development in recipient mothers.

A transgenic animal is an animal that carries a foreign gene deliberately inserted into its genome. The foreign gene is constructed in vitro using the rDNA technology. The plasmid DNA contains not only the gene of interest but also other DNA sequences, including a promoter segment (to time gene expression and target the gene to a specific tissue), enhancer sequences (to amplify gene function), and a marker gene (to detect incorporation of the DNA into the genome of the animal). The DNA construct is then incorporated into the animal’s germ-line genome by one of the two established methods: (a) injection of the rDNA (also known as a DNA construct) into the pronucleus of a fertilized ovum (Method I); and (b) injection of transformed embryonic stem cells that contain the rDNA into a blastocyst (Method II). Due to the lack of an established porcine cell line of true embryonic stem cells, only Method I (genetic modifications in somatic cells and SCNT) has been used to generate genetically engineered pigs [38]. In addition, transposable elements (transposons) [39] and retrovirus vectors [40] can be used to generate and manipulate transgenic animals.

In the first and most common method, an ovum is surgically collected shortly after its fertilization, and a rDNA (e.g., the plasmid DNA of interest) is then microinjected through a very fine needle into the pronucleus of the fertilized ovum [24]. Alternatively, an ovum receives an intracytoplasmic injection of sperm transfected with a plasmid DNA of interest [41]. The transformed ovum is developed into a blastocyst in vitro and the blastocyst is then transferred into a surrogate female for development to term. Some of the offspring (transgenic) contain the rDNA that has been integrated into their own genome. Because the foreign gene is present in both germ cells and somatic cells, it can be inherited by breeding to new progeny [24]. Note that in the case of combining cloning and gene transfer techniques, donor somatic cells can be genetically engineered via electroporation or viral vectors, followed by the production of transgenic offspring through SCNT.

In the second method, a gene is introduced into animals via embryonic stem (ES) cells. ES cells, derived from the preimplantation embryo, can both self-renew and retain pluripotential characteristics to allow for gene targeting and contribution to the germline after transfer into the early embryo [19]. Briefly, ES cells are isolated from an early embryo for culture to establish cell lines, followed by the introduction of an rDNA (e.g., the plasmid DNA of interest) into their genome through cell transfection. Stably transformed ES cells are selected and then injected into the inner cell mass of a recipient blastocyst to participate in the development of the blastocyst into an early embryo. As for the first method, the embryo that contains the recombinant gene in its genome is transferred into a surrogate female for development to term. Heterozygous transgenic offspring are mated to produce a homozygous transgenic strain.

### Advantages and applications

Transgenic animal technology allows for the introduction of a foreign gene into the germ line of an animal to establish a desirable trait (e.g., high rates of lean tissue gain and feed efficiency) and a new capacity (e.g., synthesis of a protein with nutritional applications) in a breeding line of livestock. The outcome is the successful production of transgenic animals, including pigs, mice, rats, cattle, rabbits, sheep, chickens, and fish [10]. This can complement the traditional breeding techniques to improve the efficiency of livestock production by enhancing: (a) the digestion, absorption and utilization of dietary nutrients, (b) resistance to metabolic and infectious diseases; and (c) adaptation to the living environment [18, 42, 43]. Furthermore, transgenic pigs [e.g., α-1,3-galactosyltransferase locus (*GGTA1*) knock-out swine produced through nuclear transfer by using gene-targeted fibroblasts] can provide organs for xenotransplantation in biomedicine [44].

Transgenic animals can produce nutritionally essential fatty acids [45], and amino acids, therapeutic proteins [25, 26, 31], nutritionally significant proteins [46], and enzymes to eliminate anti-nutritional factors [47]. The latter approach can improve the efficiency of nutrient utilization to reduce the number of animals on farms as well as the environmental pollution of nitrogen and phosphorus. Transgenic animals produce: (a) lysozymes that have bacteriostatic properties against mastitis-causing bacteria [48], (b) human and bovine lactoferrin proteins that have a broad-spectrum antimicrobial activity [46], and (c) vaccines (e.g., efficacious malarial vaccines) in milk [49].

As a proof-of-principle, transgenic pigs expressing human growth hormone were created over 33 years ago by Hammer et al. [24]. A few years later, transgenic pigs were generated that over-expressed bovine growth hormone or growth hormone releasing factor to increase their growth rate and feed efficiency, while reducing the content of body fat and plasma cholesterol levels [50, 51]. However, these beneficial traits were offset by negative effects, including reduced reproductive performance, the onset of diseases (e.g., arthritis, gastric ulcers, dermatitis, and renal disease) and premature death [50]. These undesired effects of transgenesis in animals occur because of an incomplete understanding of: (a) conditions (e.g., the composition of nutrients such as amino acids, glucose, minerals and vitamins in the medium) for the culture of embryos, (b) regulatory elements responsible for normal patterns of expression, (c) the site of foreign DNA integration, and (d) the physiological functions of specific gene products. Much research is warranted to address these areas of research to allow for economic and ethical production of transgenic animals.

One promising result of animal transgenesis is the production of pigs that can synthesize essential polyunsaturated fatty acids. For example, Saeki et al. [52] introduced a plant gene ∆^12^ fatty acid desaturase into the white adipose tissue of pigs. This enzyme desaturates oleic acid (C18:1, ω9) at C12 to produce linoleic acid (C18:2, ω6), a nutritionally essential polyunsaturated fatty acid in swine [4]. Linoleic acid is a precursor for the synthesis of arachidonic acid (C20:4, ω6), which is also a nutritionally essential polyunsaturated fatty acid in swine. In addition, linoleic acid is beneficial for the cardiovascular health of humans and swine. There were also transgenic pigs expressing a *C. elegans* gene for fatty acid desaturase that can convert linoleic acid into a ω3 polyunsaturated fatty acid [53]. Compared with wild-type counterparts, the transgenic pigs had greater concentrations of four ω3 polyunsaturated fatty acids: α-linolenic acid (C18:3, ω3), eicosapentaenoic acid (C20:5, ω3), docosapentaenoic acid (C22:5, ω3), and docosahexaenoic acid (C22:6, ω3). These findings are highly significant, because when transgenic pigs can synthesize ω6 and ω3 polyunsaturated fatty acids, the use of plant-source oils (e.g., soybean oil, sunflower oil, and peanut oil) and fish oil in diets can be reduced or possibly eliminated to decrease swine production costs.

Another promising outcome of the modern biotechnology is the production of transgenic pigs that express a microbial phytase in the salivary gland. A line of transgenic Yorkshire pigs (the Cassie line) was first generated to release microbial phytase in the saliva [54]. This line of pigs had an increased ability to digest feed phytate. For example, when fed typical commercial diets without supplemental phosphorous (P), transgenic boars and gilts grew and utilize feed at rates similar to those for conventional, age-matched counterparts fed the similar diets with supplemental P. In addition, transgenic barrows fed a low-P diet without supplemental P retained 25–40%, 77–91%, and 27–56% more P, respectively, during the weaning, growing, and finishing phases than conventional Yorkshire barrows fed similar diets without supplemental P. Most recently, Zhang et al. [46] produced transgenic pigs that express, in their salivary glands, both phytase and carbohydrases (xylanase plus two types of β-glucanase) based on the isolation of the genes from bacteria and fungi. The transgenic pig can start to hydrolyze phytates and non-starch polysaccharides in the mouth, and produce up to 24% less nitrogen and 44% less in wastes, compared with non-transgenic pigs fed the same diet. The quantitative differences between these studies may be due to differences in the expression levels of the transgenes and the composition of nutrients (including Ca, P, and protein) in the diets [54–56]. Potentially, pigs that express plant or microbial genes for the synthesis of nutritionally essential amino acids can permit the feeding of low-P and low-protein diets without the need for dietary supplementation with P or crystalline amino acids. One of those amino acids is threonine [57], which is low in plant-source feedstuffs relative to piglet growth [58].

### Disadvantages

The original methods of animal transgenesis (pronuclear injection and integrating viruses) had a very low efficiency, while resulting in gene silencing, poor regulation of gene expression, and large variability due to random integration of genes [19]. Another major disadvantage of the transgenic animal technology is the occurrence of high rates of prenatal and preweaning mortalities in livestock species, including swine. This may result from the random integration of genes into the host genome that results in insertational mutagenesis. For example, Zhang et al. [47] reported that after 4008 reconstructed embryos were transferred to 16 recipient sows, only 33 live piglets were born, with the efficiency of embryonic development to term being < 1%. Among the 33 live-born piglets, 25 of them were positive for a transgene, with 20 piglets having the intact transgene expression cassette. Disappointingly, only 9 transgenic pigs survived to weaning. These problems, coupled with exceedingly high costs, must be overcome before transgenic pigs are used in production agriculture. At present, transgenic livestock research focuses on nutrient utilization [52–56], disease resistance [59–64], and biomedical applications, such as xenotransplantation of organs without the α-1,3-galactosyltransferase gene that is responsible for the hyperacute rejection response [18, 19, 65, 66].

Method II for producing transgenic animals is not successful for livestock because there is no report of ES or induced pluripotent stem (iPS) cells that can endure genetic modifications and still contribute to the germ line [19]. This is a critical shortcoming of pig models or possibly the entire livestock. Genetic modification of somatic cells followed by the SCNT had been the only option to generate genetically engineered pigs carrying site-specific alterations until the direct injection of the genome editing system into developing embryos [18, 63–66].

## Gene (genome) editing to produce genetically modified animals with gene knock-out or knock-in

**Fig. 5:**
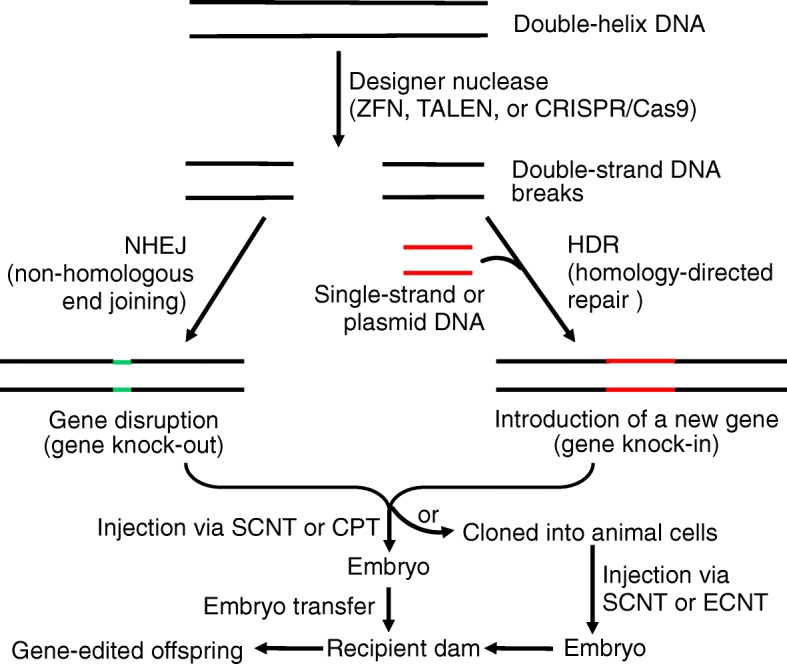
Gene (genome) editing of animals using the ZFN, TALEN or CRISPR/Cas9 technique. a designer nuclease (ZFN, TALEN or CRISPR/Cas9) cleaves a DNA molecule to generate a double strand break (DSB) at a desired genomic locus. Thereafter, one of two endogenous repair mechanisms may repair the DSB DNA: non-homologous end joining (NHEJ) and the homology-directed repair (HDR). In the NHEJ pathway, the two ends of the DSB DNA are brought together and ligated without a homologous template for repair, which often inserts or deletes nucleotides (indels) to cause gene disruption (knockout). The HDR pathway requires the provision of an exogenous DNA template along with a site-specific genome editing nuclease to repair the DSB DNA, thereby causing the knock-in of a desired sequence of DNA into the genome of an embryo or animal cells. Because of its more precise targeting of genes, CRISPR/Cas9 is gaining momentum in life sciences as the preferred editor of gene editing of livestock species

The initial methods used to generate transgenic livestock resulted in random transgene insertion [10]; therefore, new technologies are needed to enable better gene targeting with a higher efficiency in livestock. Although it is conceptually simple to deliver DNA into a fertilized egg via pronuclei injection, this method is technically challenging and the injected DNA construct is integrated randomly into the genome, resulting in unpredictable transgene expression profiles. In addition, microinjection can damage the zygote and requires expensive equipment. These short-comings can be partly overcome by the development of gene (genome) editing approach, which uses a designer nuclease (as a pair of molecular scissors) to generate a double strand break (DSB) in DNA at a desired genomic locus (Fig. 5). Thereafter, one of two endogenous repair mechanisms may repair the DSB DNA: non-homologous end joining (NHEJ) and the homology-directed repair (HDR) [38]. In the error-prone NHEJ pathway, the two ends of the DSB DNA are brought together and ligated without a homologous template for repair, which often inserts or deletes nucleotides (indels). If an indel results in a frame shift mutation, the target gene may lose function (knockout). The HDR pathway requires the provision of an exogenous DNA template along with a site-specific genome editing nuclease to repair the DSB DNA, thereby causing the knock-in of a desired sequence of DNA into the genome of an embryo or animal cells [67]. In practice, modification of a targeted gene is commonly achieved by microinjecting, into an embryo obtained by in vitro fertilization or intracytoplasmic transfer, a gene editing system that consists of a guide RNA and the Cas9 endonuclease, and, if necessary, a repair DNA template [68]. The guide RNA provides sequence specificity to target the Cas9 endonuclease to a complementary site in the genome for creating a DSB.

An earlier designer nuclease was zinc finger nuclease (ZFN; the first gene editing tool), and a subsequently discovered designer nuclease is transcription activator-like effector nuclease (TALEN, the second gene editing tool), both of which are modular proteins containing an adaptable DNA-binding domain. The ZFN method involves engineering a protein that contains both a zinc finger DNA-binding domain and a restriction endonuclease domain. The TALEN approach utilizes engineered enzymes containing a DNA-binding domain and a separate DNA-cleaving domain. In recent years, CRISPR (clustered regularly interspaced short palindromic repeats)-associated nuclease-9 (CRISPR/Cas9) has been used as a designer nuclease to provide a more efficient, more accurate, more versatile, more robust, and simpler tool in genomic engineering [19, 62]. ZFN, TALEN, or CRISPR/Cas9 components are delivered into target cells through transfection (lipid-based agents, electroporation, nucleofection, or microinjection) or bacteriophages, depending on cell type and plasmid [69–71].

TALENs and CRISPR/Cas9 were first successfully used in pigs in 2013 [72] and 2014 [59, 73], respectively. Over the past 5 years, CRISPR/Cas9 has rapidly gained momentum as the favored gene editor for livestock species. The CRISPR-Cas9 system was discovered in 2007 in bacteria (e.g., a genus of gram-positive cocci or spherical bacteria) and archaea, and is used naturally to defend against invading viruses (bacteriophages). In response to a viral infection, the bacterial CRISPR/Cas9 is guided by a short RNA fragment known as a guide RNA to snip off a piece of viral DNA, creating a DSB in its target loci [68]. The guide RNA is complementary to a segment of the genome of the targeted organism, so that the Cas9 nuclease will cleave DNA with a high degree of specificity. Of note, recognition of the target DNA by Cas9 is dependent on the presence of a short protospacer adjacent motif (PAM) sequence located directly downstream on the untargeted DNA strand [38]. Thus, the CRISPR system consists of two components (a Cas9 endonuclease and a guide RNA) as a ribonucleoprotein. Experimentally, the guide RNA can be designed using molecular biology tools in the laboratory to direct Cas9 to a specific DNA sequence for cleavage at virtually any genomic locus. The milestones for the use of CRISPR/Cas9 in producing gene-edited swine are shown in Table 2.

**Table 2:** Milestones in the use of gene editing techniques for producing gene-edited swine

Gene	Gene editor	DSB DNA repair	Route of gene injection	Year	Reference
*PPARγ*	ZFN	NHEJ	SCNT	2011	Yang et al. [74]
*LDLR*	TALEN	NHEJ	SCNT	2012	Carlson et al. [75]
*RELA*	ZFN	NHEJ	CPI	2013	Lillico et al. [72]
*RELA*	TALEN	NHEJ	CPI	2013	Lillico et al. [72]
*APC*	TALEN	HDR	SCNT	2013	Tan et al. [76]
vWF	CRISPR/Cas9	NHEJ	CPI	2014	Hai et al. [73]
*CD163*	CRISPR/Cas9	NHEJ	SCNT	2014	Whitworth et al. [59]
*CD163*	CRISPR/Cas9	NHEJ	SCNT	2016	Whitworth et al. [60]
*OTR*	CRISPR/Cas9	HDR	SCNT	2016	Lai et al. [77]
*Myostatin*	TALEN	NHEJ	SCNT	2016	Rao et al. [78]
*PERVs*	CRISPR/Cas9	NHEJ	SCNT	2017	Niu et al. [62]
*UCP1*	CRISPR/Cas9	HDR	SCNT	2017	Zheng et al. [79]
*CD163* ^*a*^	CRISPR/Cas9	NHEJ	SCNT	2017	Wells et al. [61]
*CD163*	CRISPR/Cas9	NHEJ	SCNT	2018	Yang et al. [64]
*CD163* ^*b*^	CRISPR/Cas9	NHEJ	SCNT	2018	Burkard et al. [63]

*APC* Adenomatous polyposis coli (a colon cancer gene), *CD163* Cluster of differentiation 163 (encoding for a protein that is the high affinity scavenger receptor for the hemoglobin-haptoglobin complex), *CPI* Cytoplasmic injection, *CRISPR/Cas9* Clustered regularly interspaced short palindromic repeats-associated nuclease-9, *DSB* A double strand break, *HDR* Homology-directed repair, *LDLR* Low-density lipoprotein receptor, *NHEJ* Non-homologous end joining, *OTR* Oct4-td-tomato reporter gene, *PERVs* Porcine endogenous retroviruses, *RELA* Transcription factor p65 (also known as nuclear factor NF-kappa-B p65 subunit), *SCNT* Somatic cell nuclear transfer, *TALEN* Transcription activator-like effector nuclease, *UCP1* Uncoupling protein-1, *vWF* Von Willebrand factor, and *ZFN* Zinc finger nuclease

^a^Replacement of porcine CD163 scavenger receptor cysteine-rich domain 5 with a CD163-like homolog

^b^*CD163* with exon 7 deleted

### Advantages and applications

Traditional livestock breeding is beset with such problems as long breeding cycles and limitations of genetic resources. In contrast, genome editing tools can provide more precise, more specific, more predictable and more rapid solutions to solving these problems at relatively affordable costs [38]. Thus, besides knocking out gene function, CRISPR can be employed to delete large DNA fragments from the genome of an animal. Furthermore, a gene editing technique requires fewer steps and has a higher efficiency than the previous methods of animal transgenesis. For example, studies with livestock zygotes have shown a 30% editing frequency with ZFN, TALEN and CRISPR/ Cas9 techniques [19, 72–77]. Compared to other gene silencing techniques such as RNAi and antisense RNA, CRISPR/Cas9 offers a higher efficiency, an ability to cleave methylated loci, greater ease of design, and greater flexibility [68]. It should be borne in mind that knockout of a gene provides a cleaner phenotype than its knockdown and that production of knockout pigs does not necessarily require application of a genome editing system. Several laboratories have reported success with producing gene-edited pigs, which can potentially serve as organ donors, disease models, bioreactors, inactivation of porcine endogenous retrovirus in pigs, or founder animals of genetic lines with enhanced productivity (e.g., muscle growth) or disease resistance traits [59–64]. Thus, gene editing increases successes in single-gene and multi-allelic modifications of the livestock genome, as well as in site-specific introductions of foreign genes during embryogenesis.

There are many examples for the genome editing-based production of transgenic pigs with important production and disease-resistance traits, including nutrient utilization and meat production as well as resistance to viral infections and metabolic disorders (Table 3). First, disruption of the myostatin (a negative regulator of myogenesis) gene using TALEN as an editor successfully created myostatin-knockout pigs, which exhibited a double-muscled phenotype, greater body weight, greater longissimus muscle mass, and a 100% increase in the number of muscle fibers than wild-type pigs [78]. Second, utilizing the CRISPER/Cas9 technology, Zheng et al. [79] produced pigs with a functional uncoupling protein 1 (UCP1). UCP1 is expressed in the brown adipose tissue of many animal species and is responsible for nonshivering thermogenesis, thereby playing a crucial role in protecting against cold and regulating energy homeostasis. However, modern pigs lack functional UCP1 genes and are therefore susceptible to cold stress, resulting in a high rate of neonatal mortality, and also spontaneous accumulation of a large amount of white adipose tissue in the body, leading to reduced production performance [3]. Of note, insertion of the mouse adiponectin-UCP1 gene into the porcine endogenous *UCP1* locus via the CRISPR/Cas9 as an editor can generate UCP1-knockin pigs that exhibit an improved ability to maintain body temperature, a decreased white fat mass, and an increased lean carcass yield [79]. Third, CRISPR/Cas9 gene targeting and SCNT technologies have been used to create pigs without the CD163 gene that encodes a cellular receptor for the porcine reproductive and respiratory syndrome virus-1 (PRRSV-1, also referred to as “blue ear disease” virus) [59]. Whitworth et al. [60] reported that pigs with the CD163 knock-out were fully resistant to the PRRSV-1 (European strain) and PRRSV-2 (North American strain). Similar results were observed by Burkard et al. [63] against both PRRSV-1 and PRRSV-2, and by Yang et al. [64] against a highly pathogenic PRRSV strain (belonging to the North American strain) isolated in South China. Interestingly, Wells et al. [61] found that genetically modified pigs, which were produced through the replacement of porcine CD163 scavenger receptor cysteine-rich domain 5 with a CD163-like homolog, were resistant to PRRSV-1 but not to PRRSV-2. Males and females can be used as breeding stocks to produce generations of PRRSV-resistant offspring.

**Table 3:** Production of transgenic pigs with important production and disease-resistance traits

Gene	Target tissue	Production trait	Reference
Bovine growth hormone (knock-in)	Tissues	Increases lean tissue growth and feed efficiency; reduces whole-body fat content and blood cholesterol concentration	Pursel et a1. [50] Solomon et al. [51]
Spinach ∆^12^ FAD (knock-in)	White adipose tissue	Desaturates oleic acid (18:1, ω9) at C12 to produce linoleic acid (18:2, ω6) in animals	Saeki et al. [52]
*C. elegans* FAD (knock-in)	White adipose tissue	Desaturates linoleic acid (18:2, ω6) to produce ω3 polyunsaturated fatty acids in animals	Lai et al. [53]
Microbial phytase (knock-in)	Salivary gland	Hydrolyzes phytate in plant-source ingredients; increases utilization of dietary phosphate, other minerals, and protein	Golovan et al. [54]
Phytase and other enzymes^a^ (knock-in)	Salivary gland	Hydrolyzes phytate and complex carbohydrates in plant-source ingredients; increases utilization of dietary phosphate, other minerals, and protein	Zhang et al. [47]
Myostatin (knock-out)	Skeletal muscle	Increases the number of skeletal muscle fiber, skeletal muscle mass, protein deposition, and gain:feed ratio (feed efficiency)	Rao et al. [78]
Uncoupling protein 1 (knock-in)	Tissues	Increases nonshivering thermogenesis and piglet survival; decreases the accretion of white adipose tissue; increases carcass lean tissue content	Zheng et al. [79]
CD163 (knock-out)	Tissues	Resistant to porcine reproductive and respiratory syndrome virus (PRRSV, “blue ear disease”)	Burkard et al. [63]; Wells et al.[61]; Whitworth et al. [59, 60]; Yang et al. [64]

*FAD* Fatty acid desaturase, *GH* Growth hormone

^a^Xylanase plus two types of β-glucanase (bacterial and fungal genes)

### Disadvantages

Although the ZFN method provided the first breakthrough in site-specific gene editing, it has some limitations, such as off-target cutting of the DNA, cytotoxicity, expensive, time-consuming, low efficiency (thus only one genomic edit at a time), and technical challenges to prepare effective ZFN tools [38]. Compared with the ZFN editor, the TALEN technique is more flexible in genetic engineering because its DNA-binding domain can target a wider range of DNA sequences. Although the TALEN editor is easier to design than the ZFN, the TALEN method is expensive and technically difficult when the goal is to simultaneously make multiple edits to the genome [68]. In addition, delivering the gene-editing Cas9 directly to embryos by microinjection remains a challenging process, and microinjection itself may damage the embryos. Compared to the ZFN and TALEN, CRISPR/Cas9 is known to have a higher frequency of off-target effects [80]. Another major obstacle to the use of the CRISPR/Cas9 technology for generating gene-edited animals is the problem of mosaicism (the presence of more than one genotype in one individual) that is common in founder animals [68]. Furthermore, for all currently available gene-editing methods, the rates of prenatal mortality in gene-edited fetuses are much greater than those for control fetuses. To date, the efficiency of gene editing in livestock, including swine, remains suboptimal. The procedures for gene editing should be easier and cheaper, so that more producers can utilize this innovative technique on their own farms for improving animal breeding.

## Biotechnology for understanding antibiotic resistance in animals and developing alternatives to in-feed antibiotics for swine feeding

Since the discovery of penicillin in 1928, antibiotics have been used to treat bacterial infections in humans and animals. Since the 1950s, sub-therapeutic levels of antibiotics have been included in conventional diets to improve the growth performance of swine and poultry. However, due to the development and spread of bacteria resistant to antibiotics, feed antibiotics have been banned in many countries (e.g., the European union) and are being phased out in some major swine-producing nations (e.g., the U.S. and China). Some bacteria are resistant to one class of antibiotics, and others are resistant to multiple antibiotics, thereby posing a serious global health concern [81]. For ensuring the optimal efficacy of antibiotics in treating bacterial infections in animals and humans, there is increasing concern worldwide over antimicrobial resistance (AMR), which can be defined as the ability of bacteria to resist the effects of an antimicrobial (e.g., antibiotics).

The antimicrobial resistance genes in bacteria can be inherited from mother to daughter cells by division, as well as from one strain to another via plasmid transfer. Interestingly, the plasmids (small DNA molecules which are independent from the chromosomal DNAs) in bacteria often carry information that may benefit their own survival through resistance to antibiotics produced by themselves or by other organisms in their environment [82]. In 2007, an analysis of a colistin-resistant *E. coli* isolate in China revealed a plasmid with 19 antibiotic resistance genes. When a troublesome antibiotic is not used for a prolonged period of time, resistance levels in bacteria decrease, but can increase again when the antibiotic is used again [83]. Thus, there is an urgent need to identify new alternatives to feed antibiotics in swine production worldwide. This can be greatly facilitated by the use of biotechnology to understand how AMR occurs.

**Fig. 6:**
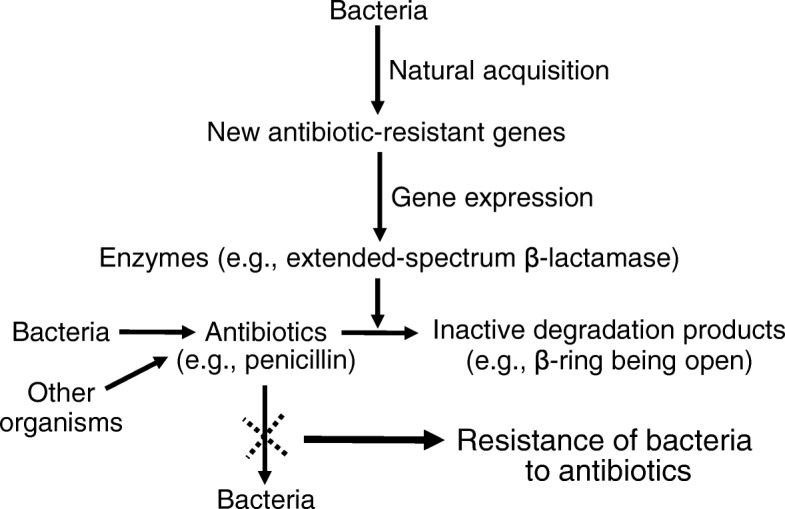
Mechanisms responsible for the development of antimicrobial resistance in bacteria. Bacteria naturally acquire new genes (including antimicrobial-resistant genes) to survive in a new environment or host. The antimicrobial-resistant genes produce enzymes (e.g., extended-spectrum β-lactamase in *E. coli*) to destroy or inactivate antibiotics. For example, penicillin-resistant bacteria synthesize β-lactamase, which breaks down the β-lactam ring of penicillin to an inactive degradation product. Through this mechanism, the bacteria cannot be killed by penicillin, leading to antimicrobial resistance in infected animals and humans. The sign (X) denotes an inability to kill bacteria

**Fig. 7:**
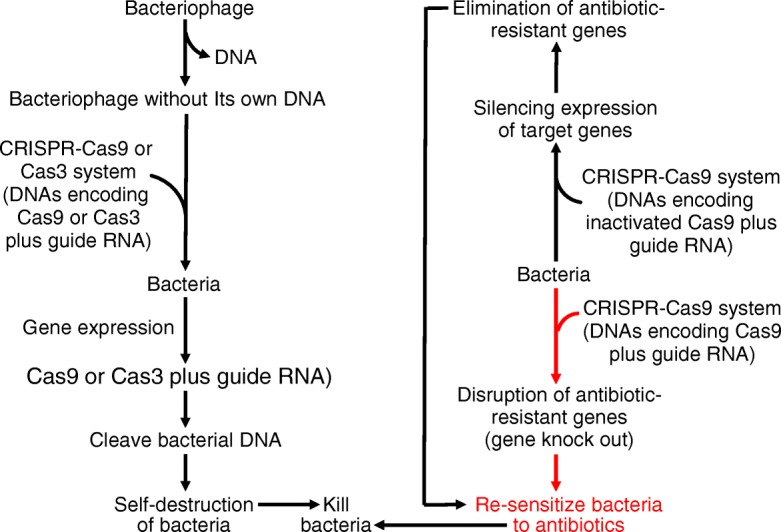
Utilization of the CRISPR system as a new alternative to antibiotics. The CRISPR-Cas system has an ability to selectively target specific DNA sequences and, therefore, can easily distinguish between pathogenic or commensal bacterial species. Bacteriophages can be utilized to deliver the CRISPR-Cas cargo into bacteria through receiving either a designed DNA that encodes a guide RNA and Cas9 or a guide RNA and Cas3 to cut bacterial DNA molecules at multiple sites, causing self-destruction of the bacteria. Alternatively, the CRISPR-Cas9 system can be utilized to knock out genes responsible for antimicrobial resistance and re-sensitize the multidrug resistant bacteria, so that they will be killed by antibiotics. Finally, a CRISPR-Cas9 system can be constructed as a CRISPR interference (CRISPRi) plasmid vector that carries a DNA sequence for inactivated Cas9 and a guide RNA to silence the expression of membrane-bound virulent proteins and antibiotic-resistant genes

Much evidence shows that bacteria naturally acquire new genes (including antimicrobial-resistant genes) to survive in a new environment or host [84]. The antimicrobial-resistant genes produce enzymes (e.g., extended-spectrum β-lactamase in *E. coli) to d*estroy or inactivate antibiotics (Fig. 6). For example, penicillin-resistant bacteria (e.g., *Staphylococci aureus* and *E. coli*) synthesize β-lactamase, which breaks down the β-lactam ring of penicillin to an inactive substance. Through this mechanism, the bacteria cannot be killed by penicillin, leading to AMR. Now, CRISPR-based methods are being developed to kill antibiotic-resistant bacteria, because CRISPR-Cas possesses its ability to selectively target specific DNA sequences and, therefore, easily distinguish between pathogenic or commensal bacterial species [82, 85]. Of particular note, bacteriophages (generally safe for animals and humans) have been used to deliver the CRISPR-Cas system into bacteria [84]. For example, bacteriophages without their own DNA receive a designed DNA that encodes a guide RNA and Cas9 [70]. The bacteriophages are then transfected into antibiotic-resistant bacteria (e.g., *Clostridium difficile)*, where Cas9 is guided by the guide RNA to cut the bacterial DNA at specific sites, triggering the bacteria to self-destruct (Fig. 7). Similarly, a CRISPR-Cas3 system has been delivered via bacteriophages into both Gram-positive and Gram-negative bacteria to cut DNA molecules at multiple sites, thereby driving programmed cell death [86]. Furthermore, Kim et al. [82] used the CRISPR-Cas9 system to knock out genes responsible for AMR and re-sensitize the multidrug resistant bacteria, so that they are killed by antibiotics. Finally, the CRISPR-Cas9 system, which is constructed as a CRISPR interference (CRISPRi) plasmid vector carrying a DNA sequence for inactivated Cas9 and a guide RNA, has been used to eliminate membrane-bound virulent proteins (e.g., coagulase A and enterotoxin type C) and antibiotic-resistant genes (e.g., β-lactamases) in *Staphylococci aureus* (Gram-positive bacteria) [87]. In this method, two domains in the inactivated Cas9 are mutated, and this protein has only a DNA binding activity, but cannot cleave DNA. The binding of the inactivated Cas9 (dCas9) interferes with gene expression in bacteria by preventing their transcription machinery from accessing the target gene, thereby silencing its expression. Thus, the CRISPR-Cas9 technologies, which involve bacteriophages or plasmids, hold promise for killing bacteria and removing enzymes from bacteria, including antimicrobial-resistant bacteria, in the gastrointestinal tract of animals (Table 4). A practical application of this technology would be to mitigate AMR and develop alternatives to in-feed antibiotics in swine production. Such a genetic engineering approach, along with feedstuff fermentation and the preparation of antimicrobial peptides from feed proteins [88], is expected to maximize the efficiency of nutrient utilization and sustain the pork industry worldwide.

**Table 4:** Utilization of the CRISPR-Cas9 system as new alternatives to the use of antibiotics

System	Vector	Target gene	Antibiotic-resistant bacteria	Reference
CRISPR-Cas9	Bacteriophages	Multiple DNA sites (self-destruction)	*Clostridium difficile*	Bikard et al. [70]
CRISPR-Cas3	Bacteriophages	Multiple DNA sites (self-destruction)	Gram-positive and negative bacteria	Reardon [86]
CRISPR-Cas9	Plasmids	Antibiotic-resistant genes (knock out)	*Escherichia coli*	Kim et al. [82]
CRISPR-Cas9	CRISPRi plasmids	Antibiotic-resistant genes and membrane proteins (silencing gene expression)	Staphylococci aureus and other Gram-positive bacteria	Sato’o et al. [87]; Greene [84]

*CRISPR/Cas3* Clustered regularly interspaced short palindromic repeats-associated nuclease-3, *CRISPR/Cas9* Clustered regularly interspaced short palindromic repeats-associated nuclease-9, *CRISPRi* CRISPR interference

## Conclusion

Demands for high-quality meat protein drives the global pork industry to increase its productivity, while reducing carbon footprints and waste excretion. To achieve this goal, there has been revolutionary progress in animal biotechnology over the past 35 years to produce recombinant proteins (including enzymes) and organic nutrients (including amino acids and vitamins), clones of swine, and genetically modified pigs for both biomedical and agricultural purposes. In recent years, gene (genome) editing technologies based on ZFN, TALEN and CRISPR/Cas9 as editors have become available to delete, insert, or modify the genome of animals (including pigs) and bacteria at the specific sites of DNA sequences. Compared to the ZFN and TALEN editors, CRISPR/Cas9 offers greater ease of design and greater flexibility in genetic engineering, but has a higher frequency of off-target effects. Thus, with continuous improvements, this biotechnology holds great promise in conserving the diverse breeds of swine, augmenting feed efficiency and pork production, and developing alternatives to antibiotics in the future.

## Abbreviations

AMR: Antimicrobial resistance
CRISPR/Cas9: Clustered regularly interspaced short palindromic repeats-associated nuclease-9
CRISPRi: Clustered regularly interspaced short palindromic repeats interference
DSB: A double strand break
ECNT: Embryonic cell nuclear transfer
ES: Embryonic stem
HDR: Homology-directed repair
NHEJ: Non-homologous end joining
PRRSV-1: Porcine reproductive and respiratory syndrome virus-1
QTL: Quantitative trait locus
rDNA: Recombinant DNA
SCNT: Somatic cell nuclear transfer
SNPs: Single nucleotide polymorphisms
TALEN: Transcription activator-like effector nuclease
UCP1: Uncoupling protein 1
ZFN: Zinc finger nuclease
